# The Quantum Efficiency Roll-Off Effect in Near-Infrared Organic Electroluminescent Devices with Iridium Complexes Emitters

**DOI:** 10.3390/ma13081855

**Published:** 2020-04-15

**Authors:** Wojciech Mróz, Sagar Kesarkar, Alberto Bossi, Daniel Pelczarski, Piotr Grygiel, Waldemar Stampor

**Affiliations:** 1 Institute of Macromolecules’ Study (ISMAC), National Research Council (CNR), Via E. Bassini 15, 20133 Milan, Italy; Wojciech.Mroz@iit.it; 2 Centre for Nano Science and Technology (CNST@PoliMi), Italian Institute of Technology (IIT), Via Pascoli 70/3, 20133 Milan, Italy; 3 Istituto di Scienze e Tecnologie Chimiche “G. Natta”, Consiglio Nazionale delle Ricerche (CNR-SCITEC), Via Fantoli 16/15, 20138 Milano and SmartMatLab Center, Via C. Golgi 19, 20133 Milano, Italy; sagar.kesarkar2@unibo.it (S.K.); alberto.bossi@cnr.it (A.B.); 4 Department of Physics of Electronic Phenomena, Faculty of Applied Physics and Mathematics, Gdańsk University of Technology, Narutowicza 11/12, 80–233 Gdańsk, Poland; daniel.pelczarski@pg.edu.pl (D.P.); waldemar.stampor@pg.edu.pl (W.S.)

**Keywords:** OLEDs, infrared emitters, iridium complex, quantum efficiency roll-off, triplet-triplet annihilation

## Abstract

The electroluminescence quantum efficiency roll-off in iridium(III)-based complexes, namely Ir(iqbt)_2_(dpm) and Ir(iqbt)_3_ (iqbt = 1 (benzo[b]thiophen-2-yl)-isoquinolinate, dpm = 2,2,6,6-tetramethyl-3,5-heptanedionate) utilized as near-infrared emitters in organic light emitting diodes with remarkable external quantum efficiencies, up to circa 3%, 1.5% and 1%, are measured and analyzed. With a 5–6 weight% of emitters embedded in a host matrix, the double-layer solution-processed structure as well as analogous three-layer one extended by a hole-conducting film are investigated. The triplet-polaron, the Onsager electron-hole pair dissociation and the triplet-triplet annihilation approaches were used to reproduce the experimental data. The mutual annihilation of triplets in iridium emitters was identified as prevailingly controlling the moderate roll-off, with the interaction between those of iridium emitters and host matrixes found as being less probable. Following the fitting procedure, the relevant rate constant was estimated to be $\left(0.5-12\right)\times 10^{-12}$ cm^3^/s, values considered to be rather too high for disordered organic systems, which was assigned to the simplicity of the applied model. A coexistence of some other mechanisms is therefore inferred, ones, however, with a less significant contribution to the overall emission quenching.

## 1. Introduction

Near-infrared (NIR) organic light emitting diodes (OLEDs) have gained wide scientific attention during the last decades since they are expected to be successfully applied in medical, security and telecommunication sectors, e.g., for intracellular imaging, night-vision and fiber optic networks [1]. The electroluminescence (EL) quantum efficiency (QE) of organic devices has, however, been progressively and significantly reduced due to the narrowing energy gap of the emitters from the visible to the NIR-region of the electromagnetic spectrum as a result of the energy-gap-law [2]. In addition, electroluminescence QEs are considerably reduced due the high current densities and electric field strengths applied to operate the OLEDs [3]. The optimal solutions regarding the fabrication techniques, components for emitting layers as well as structure of devices to limit this efficiency roll-off effect are therefore the subject of extensive investigations. As a result, numerous phosphorescent organometallic compounds based on Ir, Pt and rare earth metal ions have recently been proposed and studied to achieve higher electroluminescence QEs. In fact, in comparison to pure organic emitters, the presence of heavy metal atoms does significantly enhance the radiative decay of electrogenerated triplet excitons which is a result of strong spin-orbit coupling. This provides a beneficial effect on exciton harvesting and the OLED performance. In particular, iridium(III) NIR-emitting complexes have been a subject of interest due to the short lifetimes of excited states [4,5]. Particularly, the cationic complexes, [Ir(pbq-g)_2_(Bphen)]^+^PF6^−^ and [Ir(mpbqx-g)_2_(Bphen)]^+^PF_6_^−^ (pbq-g = phenylbenzo[g]quinoline, mpbqx-g = 2-methyl-3-phenylbenzo(g)quinoxaline, Bphen = 4,7-diphenyl-1,10-phenanthroline) were synthesized and applied to fabricate the solution-processed OLEDs. Those devices, emitting in the wavelength range of 690−850 nm, exhibited QEs of 0.67% and 0.61% accompanied by a comparatively small roll-off effect [6]. Later on, a QE of 2.2% in the 750−800 nm range was reported of devices employing charge-neutral iridium(III) acetylacetonate, namely an Ir(mpbqx-*g*)_2_acac complex (mpbqx-g = bis(2-methyl-3-phenylbenzo(g)quinoxaline-N,C’) doped in a bipolar gallium complex [7]. For OLEDs based on solution-processable, emitting complexes with fluorenyl or thienyl appendices at the peripheral side of the diphenylquinoxaline (dpqx) ligands, Ir(fldpqx)_2_(acac) and Ir(thdpqx)_2_(acac), QEs as high as 5.7% (with an emission peak at 690 nm) and 3.4% (with an emission peak at 702 nm) were respectively achieved [8]. In these two papers the minor efficiency roll-off was also detected. More recently, photoluminescence quantum yields of up to 16% in the 680–850 nm range have been reported for iridium(III) complexes based on the 1-(benzo[b]thiophen-2-yl)-isoquinolinate (iqbt) cyclometalated emissive ligand, namely Ir(iqbt)_2_(dpm), Ir(iqbt)_2_(tta) and Ir(iqbt)_2_(dtdk), where dpm = 2,2,6,6-tetramethyl-3,5-heptanedionate, tta = 2-thienoyltrifluoroacetonate and dtdk = 1,3-di(thiophen-2-yl)propane-1,3-dionate [9,10]. Those compounds made it possible to obtain electroluminescence QE values of up to 3% from the solution-processed sandwich-type structures, characterized by a minor QE roll-off. More recently, a small roll-off effect and QEs of up to 5.2% (with an emission maximum at 824 nm) as well as even 17.3% (with an emission maximum at 765 nm) for devices containing, respectively, Ir(dtbpa)_3_(dtbpa = 1,4-di(thiophen-2-yl)benzo(g)phthalazine) and Ir(Ftbpa)_3_(Ftbpa = 1-(2,4-bis(trifluoromethyl)phenyl)-4-(thiophen-2-yl)-benzo(g)phthalazine) emitters were recorded [11]. An iridium complex grafting a hole-transporting triphenylamine (TPA) unit onto a (t-BuPyrPyTPA)_2_Ir(acac) cyclometalated ligand was in turn applied to achieve an OLED QE of 0.56% with an emission peak at 697 nm successfully synthesized and characterized [12]. As for other metals, a variety of applications of emitters containing platinum, lanthanide, osmium, as well as phthalocyanine complexes, are described in the literature (see e.g., [13]) as materials for the fabrication of promising NIR OLEDs.

The QE roll-off effect may be essentially ascribed to the mechanisms of triplet-triplet annihilation (TTA), triplet-charge carrier (polaron) quenching (Tq) and electric-field induced dissociation of electron-hole (e-h) pairs [3]. In the present paper, we analyze the QE roll-off effect resulting from high current intensities exceeding 1 mA/cm^2^ and electric field strengths of the order of 10^6^ V/cm, as applied to three types of NIR-OLEDs based on the Ir(iqbt)_2_(dpm) as well as Ir(iqbt)_3_ emitters. The current density-voltage characteristics, as well as the current density-external quantum efficiency (EQE) dependencies of the devices, were recorded, the latter ones fitted using the curves representing the TTA, Tq and the Onsager models in order to determine the values of the relevant rate constants.

## 2. Experimental Section

**Figure 1:**
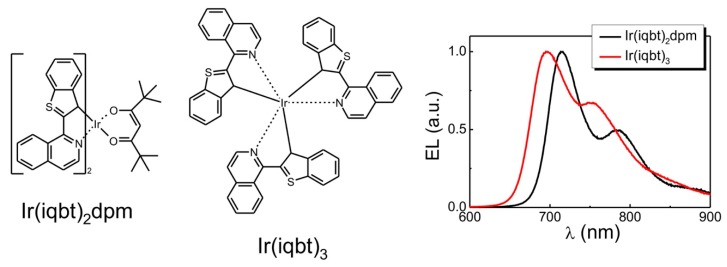
Chemical structures of the NIR-emitting iridium complexes and their EL spectra.

**Figure 2:**
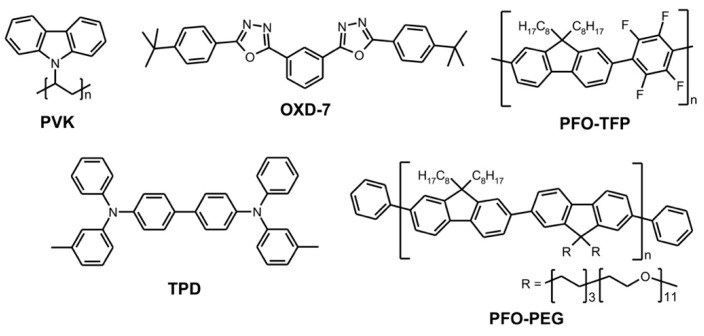
Chemical structures of compounds used for the fabrication of the devices.

In the present study, two types of OLEDs were investigated, with active layers being a part of two-layer, as well as three-layer, sandwich structures. The NIR-emitting complexes, Ir(iqbt)_2_(dpm) and Ir(iqbt)_3_, were synthesized according to the methods reported in the papers [10,14], and their chemical structures are shown in Figure 1. The two compounds have been selected as the OLED emitters since, according to previous studies [10,14], both of them exhibit an efficient photoluminescence quantum yield together with reversible redox features (i.e., reversible charge transfer behavior), energetically similar HOMO (Highest Occupied Molecular Orbital) and LUMO (Lowest Unoccupied Molecular Orbital) levels, and triplet emissive states localized on the iqbt cyclometalated ligand. Nevertheless, they differ in molecular symmetry, sterical hindrance and the presence of a solubilizing group represented by the dpm ancillary ligand. This would allow for a comparative analysis of quenching mechanisms, given that the energy landscapes of these emitters are similar. Figure 1 also shows the electroluminescence emission spectra of both compounds as measured in a range of low electric fields for the two-layer OLED systems (see further part of this section). It should be noted that these spectra remain well-correlated with those determined at 298 K in a degassed CH_2_Cl_2_ solution, not displayed here (confer [10]). The EL curves reveal a structured phosphorescence emission with distinct maxima at circa 695 nm and 715 nm, respectively for Ir(iqbt)_3_ and Ir(iqbt)_2_(dpm), which are followed by a less pronounced vibrational progression at circa 753 nm and 785 nm, hence covering the NIR, 680–900 nm, range of the electromagnetic spectrum. The light output-current-voltage (L-I-V) measurements were performed on sandwich-type devices utilizing ITO (indium tin oxide) glasses with deposited 50 nm-thick films of PEDOT:PSS (poly(3,4-ethylenedioxythiophene)-poly(styrenesulfonate)) to form the anodes, and Ba/Al (7/100 nm) vacuum-evaporated cathodes. Two types of OLEDs were investigated, the first (double-layer) one containing 65% PVK (polyvinylcarbazole): 30% OXD7: 5% Ir(iqbt)_2_dpm or 5% Ir(iqbt)_3_ mixtures applied to form the circa 200 nm-thick emitting film, with the PVK and OXD7 (1,3-bis(5-(4-tert-butylphenyl)-1,3,4-oxadiazol-2-yl)benzene) blend building the host matrix in which the phosphorescent molecules were embedded. In the second type, a 64% PFO-TFP: 30% TPD: 6% Ir(iqbt)_3_, circa 80 nm-thick active layer was used, with PFO-TFP (polydioctylfluorene-trifluoperazine) and TPD (N,N′-bis(3-methylphenyl)-N,N′- diphenylbenzidine) applied as the matrix, together with a hole-transporting PVK film of 45 nm-thickness to form a three-layer device. Apart from carrier transporting properties [15], such a usage makes advantage of PVK in preventing excitons from quenching by the ITO/PEDOT:PSS electrode, as well as in increasing the distance between the emitter and the anode, hence reducing the generation of plasmons in this region [16]. Once combined, the hole transporter TPD with the PFO-TFP exhibiting electron transporting properties would make the host matrix better suited for emitter dopant. For a further enhancement of performance, 20-nm thick PFO-PEG (PEG = poly(ethylene glycol)) electron injecting layers were utilized in structures of both types. The chemical structures of the relevant compounds are depicted in Figure 2.

Most compounds that were used were commercially available: (i) PEDOT:PSS, Clevios P VP AI 4083, Heraeus, Hanau, Germany, (ii) PVK, Sigma-Aldrich, St. Louis, MO, USA, (iii) OXD7, LumTec, New Taipei City, Taiwan and (iv) TPD, Sigma-Aldrich, St. Louis, MO, USA, whereas PFO-TFP and PFO-PEG were synthesized according to the procedures from [17] and [18], respectively. Note that PVK with a molecular weight of $1\times 10^{6}$ g/mol was used as a hole transporting layer in the three-layer device. As a host in the double-layer systems, PVK with a molecular weight of $\left(25-50\right)\times 10^{3}$ g/mol, was utilized. For more chemical aspects and the strategy for the selection of emitting materials, we refer to the study [10], as they are not dealt with in the present report.

**Figure 3:**
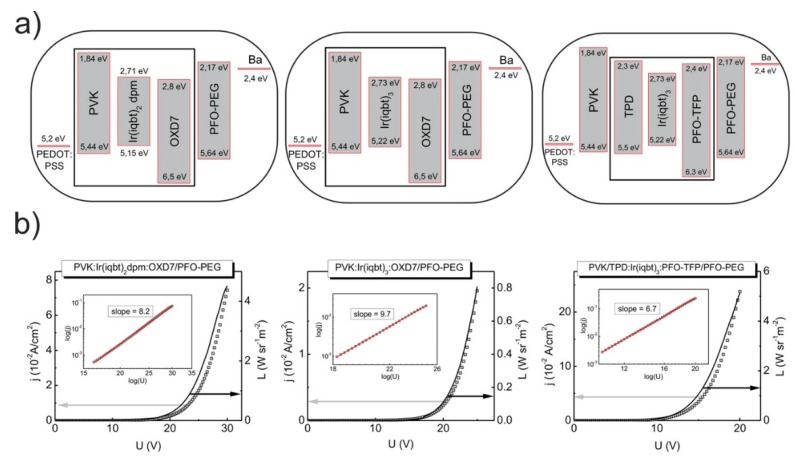
(**a**) The HOMO and LUMO energy diagrams of the double- and three-layer NIR OLEDs as taken from [10] and references therein, and (**b**) their current density-voltage-radiance (j−U−L) characteristics. Inserts: the double-logarithmic j−U characteristics with the values of the trap distribution parameter for emitting materials.

It should be emphasized here that the usage of the PEG-derivative of PFO makes it possible, by introducing a constant dipole moment between the cathode and organic emitter, to lower the potential barrier between them. Indeed, the barium work function of 2.4 eV remains close to the LUMO level of PFO-PEG that is located at 2.5 eV. Next, the PFO-PEG derivative is ethanol-soluble. Therefore, the films of relevant materials that are soluble in other (orthogonal) organic solvents can easily be coated with a PFO-PEG layer while preparing the stack structures. With the described structures, the favorable mutual position of corresponding HOMO and LUMO levels has been achieved, which is seen from the diagrams in Figure 3a. It is worth noting that all the materials and device architectures of this paper were carefully chosen as providing clear current density-external quantum efficiency dependencies to ensure a reliable modeling of the regarded physical processes.

The device preparation processes, as well the experimental procedures, were performed in nitrogen atmosphere using a M-Braun glovebox system. For all organic compounds, the simple spin coating technique could be utilized to build up the stack layers. In particular, the light emitting layers for two-layer devices were fabricated using a 15 mg/mL solution of relevant compounds in nitrogen-degassed tetrahydrofurane with a spin-coater rotating at 600 rpm. As for the measuring techniques, the EL spectra were recorded at room temperature under a constant bias applied to the devices using a Spex CCD detector (Spex, Jobin-Yvon, Palaiseau, France) cooled by liquid nitrogen, whereas the current–voltage (I–V) device characterization was performed with a Keithley 2602 source meter (Keithley, Cleveland, OH, USA). The EQEs were determined by recording the OLED forward-direction emissions, assuming the Lambertian intensity profile. The calibrated Si photodiode (OSI Optoelectronics, Hawthorne, CA, USA) was used as a detector of the radiant flux.

## 3. Results and Discussion

In what follows we discuss the influence of the current density (or electric field intensity) on the values of EQE for all investigated devices. To rationalize the EQE experimental outcomes, it was assumed that the triplet exciton recombination processes in the devices do prevailingly occur with the participation of the emissive iridium complex molecules (see the energy diagrams in Figure 3a and [10]). In order to identify the mechanisms of the EQE roll-off, the fitting procedures of the experimental data were performed, with the formulas for following the theoretical approaches: (i) the triplet-triplet annihilation (TTA) [19], (ii) the triplet-polaron quenching (Tq) [20] and (iii) the Onsager model of the electron-hole (e-h) pair dissociation process [21], i.e., the electric field-induced quenching of emitting states.

In the TTA model, collisions of moving triplet excitons of the first-excited-state, $T_{1}$, result in encounter complexes of a certain multiplicity, according to the spin conservation rules. Indeed, the TTA-resulting excitons do represent nine possible spin combinations. Particularly, one spin-zero state, three triplet states of total spin-one and five quintet ones with the total spin of two (denoted here by the symbol (T1…T1) 1,3,5 in Equation (1)) can be created upon the collision of two triplet states with randomly-oriented spins. Importantly, the quintet states are quite energetically inaccessible as their creation requires a simultaneous excitation of two electrons [22]:

(1) T1+T1⟷(T1…T1) 1,3,5→γTT{→25%Sn+S0→S1+S0(→γISCT1+S0)→75%Tn+S0→T1+S0

Following Equation (1), two further processes regarding the $\left(T_{1}\ldots T_{1}\right)$ complexes are then possible as characterized by the bimolecular rate constant, $\gamma_{\mathrm{TT}}.$ In particular, regarding the singlet channel path of the reaction scheme, the 25%-probability-transformations occur into hot singlet states, $S_{n}$, together with the molecules in the ground singlet, $S_{0}$, state. The hot $S_{n}$-states do decay in the next steps to the relaxed first-level-excitations, $S_{1}$. In the case of phosphorescent emitters, the $S_{1}$-excitons then change their multiplicity to become the $T_{1}$ ones via the process of intersystem crossing with the relevant rate constant, $\gamma_{\mathrm{ISC}}$. In turn, following the triplet channel path of the reaction of Equation (1), with a probability of 75%, the hot $T_{n}$ triplet states are produced as accompanied by the ground-state, $S_{0}$, molecules. The relaxation of $T_{n}$-excitons is the following process, yielding the first $T_{1}$-excited states. To sum up, due to the TTA mechanism from an initial pair of triplet excitons, one triplet state disappears, which decreases the population of emitting states in the relevant OLEDs.

It should be noted here that the diffusion-controlled dissociation of the $\left(T_{1}\ldots T_{1}\right)$ pair states may also occur as an alternative to the processes described above, which actually represents the back-scattering of triplet excitons without further results, and the ratio of the dissociation to annihilation rate constants is electric-field-sensitive. Next, according to Equation (1), the population of triplet excitons during electrical excitation does significantly exceed that of singlet ones. Therefore, the interaction of triplet states is usually expected to dominate in phosphorescent OLEDs.

As for the mathematical description of the TTA model, the steady-state solution of equations for triplet exciton concentrations with and without the annihilation process is regarded, and one obtains [19]:

(2) $$\frac{\mathrm{EQE}}{{\mathrm{EQE}}_{0}}=\frac{j_{0}}{4j}\left(\sqrt{1+\frac{8j}{j_{0}}}-1\right)$$

with

(3) $$j_{0}=\frac{4\mathrm{ew}}{\gamma_{\mathrm{TT}}\tau^{2}}$$

Here, EQE and ${\mathrm{EQE}}_{0}$ denote the external quantum efficiencies with and without the annihilation of excitons, respectively, j–the device electric current density, $j_{0}$–the current density at which $\mathrm{EQE}={\mathrm{EQE}}_{0}/2$, e stands for the elementary charge, w–the thickness of the recombination zone, and $\tau ={\left(k_{r}+k_{\mathrm{nr}}\right)}^{-1}$ is the exciton lifetime (with, respectively, $k_{r}$ and $k_{\mathrm{nr}}$–the exciton radiative and non-radiative decay rate constants).

In the Tq approach, the interaction between triplet excitons and charge carriers, q (particles with ½-spin or doublets), is considered. One shall, however, note that the presence of trapped carriers is essential for this mechanism, since their population greatly dominates that of free electrons/holes in an organic material. Thus, during the quenching process, encounter complexes of triplet states and trapped carriers with a doublet or quartet multiplicity as allowed from available six-spin orientations (denoted as (T1…q) 2,4 in Equation (4)) are produced.

(4) T1+q⟷(T1…q) 2,4→γTqS0+q∗

In the next step, at the expense of the energy of triplet states, the molecules in the ground singlet state are generated (with the corresponding second-order rate constant, $\gamma_{\mathrm{Tq}}$) together with relevant hot carriers, $q^{*}$. The charge carriers can in turn relax in their traps or become the free ones. Therefore, due to the Tq quenching, the population of emitting triplet excitons becomes reduced, similarly to the TTA process.

As in the case of the TTA mechanism, the mathematical descriptions of Tq quenching can be obtained by the solution of steady-state equations for triplet exciton concentrations with and without the Tq process involved. Hence, the ratio of the corresponding quantum efficiencies is given by the formula:

(5) $$\frac{\mathrm{EQE}}{{\mathrm{EQE}}_{0}}={\left(1+\gamma_{\mathrm{Tq}}{\tau n}_{t}\right)}^{-1}$$

where $n_{t}$ is the concentration of trapped charge carriers [3]. For the analysis of the experimental data, Equation (3) should be, however, rewritten to include the measured quantities. This can be done assuming that the given OLED does operate under conditions of a volume-controlled current [19,23], that is with an Ohmic injection of carriers, the carrier non-diffusive transport in the presence of an exponential trap distribution [3]. Therefore, the $n_{t}$ should be approximately proportional to the voltage, U, applied to the structure [21]:

(6) $$n_{t}=\mathrm{aU}$$

and, on the other hand, in the given conditions:

(7) $$j={\mathrm{AU}}^{l+1}$$

where a and A are constants, and l stands for the dimensionless parameter describing the distribution of traps in the emitting material. Thus, Equation (5) takes the form of:

(8) $$\frac{\mathrm{EQE}}{{\mathrm{EQE}}_{0}}={\left[1+\propto j^{1/l+1}\right]}^{-1}$$

with ∝ being a constant proportional to $\gamma_{\mathrm{Tq}}\tau$ [19]. Hence, the value of parameter l is here determined from the experimental current-voltage (or current-electric field intensity) characteristics of the investigated OLEDs.

Regarding the Onsager approach, charge carriers injected into an OLED emitter do undergo the bimolecular recombination mechanism, hence with an intermediate step in which correlated electron-hole pairs (e−h), as bound by Coulombic forces, of singlet, (e−h) 1, as well as triplet, (e−h) 3, multiplicity are produced. Note that, due to the large Coulombic interaction radius in low-mobility materials, the (e−h)-intrapair distance is of one or several lattice constants. Next, since the creation of the pairs is a statistically random event, the population of singlet pairs is three times larger than that of triplet ones. As for the (e−h) 1 pairs, their recombination to form the first-excited singlet states, $S_{1}$, can occur, this being followed in phosphorescent emitters by the formation of the triplet excitons, $T_{1}$, via the process of intersystem crossing (ISC). Alternatively, the ISC-conversion, (e−h) 1→(e−h) 3, is possible with the conversion of triplet pairs again to the $T_{1}$-excitons. The dissociation of electron-hole pairs is, however, electric-field assisted. Therefore, assuming the dominant role of such a mechanism in the operation of an OLED, the roll-off effect is supposed to be tractable in terms of the Onsager model for the recombination of (e−h) pairs, with the relevant approximating formula being:

(9) R=1−Ω(F)

where R stands for the probability of exciton formation, and Ω–the probability of the e-h pair dissociation (F is the strength of the electric field applied to the device), given by the expression [24,25]:

(10) $$\Omega \left(F\right)=1-\frac{k_{B}T}{{\mathrm{er}}_{0}F}\sum_{m=1}^{\infty }P\left(m,\frac{r_{c}}{r_{0}}\right)P\left(m,\frac{{\mathrm{er}}_{0}F}{k_{B}T}\right).$$

Here, $k_{B}$ is the Boltzmann constant, $r_{0}$–the mean intrapair distance, $r_{c}=187\;Å$ (for a temperature T=298 K and relative electric permittivity of an organic solid $\varepsilon_{r}=3$)–the Onsager radius, i.e., the distance at which the Coulombic electron-hole interaction in a pair is equal to the energy of $k_{B}T$. The symbol P(m,x) stands for the incomplete gamma function of the integral order m. Note that the calculations of the dissociation probability based on Equation (10) are relatively simple, usually yielding satisfactory results in a wide range of electric field strengths. Nevertheless, the Ω(F) can also be determined using the Sano–Tachiya–Noolandi–Hong approximation [26,27], in which, contrary to the Onsager one, the final recombination of carriers proceeds with a finite velocity and on a sphere of a finite radius, but at the expense of fairly sophisticated and tedious mathematical manipulations (examples of calculations comparing both formalisms are given in our previous paper [28]).

**Figure 4:**
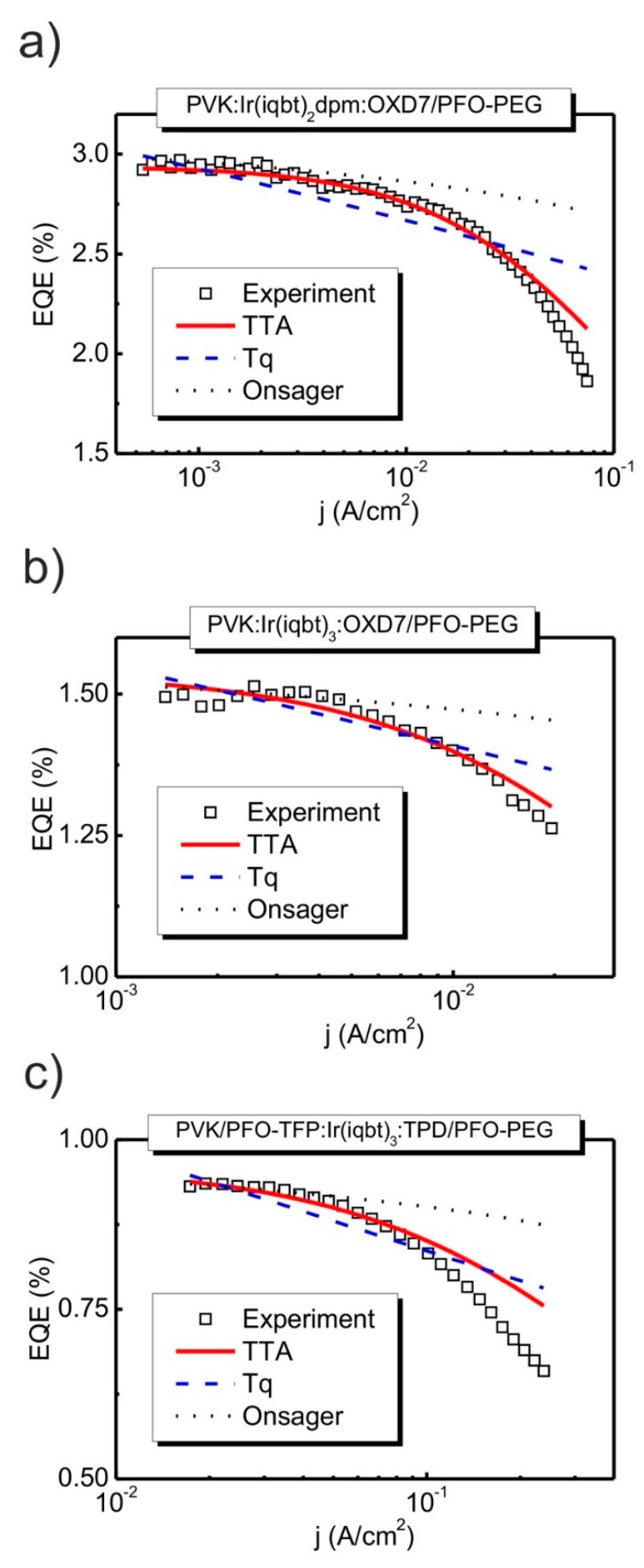
The EQE experimental data (squares) for (**a**,**b**) double-layer and (**c**) three-layer NIR OLEDs, as compared to the curves based on the TTA, Tq and the Onsager models of the roll-off effect (lines). The calculations for the Onsager approach were performed assuming $r_{0}/r_{c}=0.1$, i.e., $r_{0}$ = 1.9 nm for T=298 K and $\varepsilon_{r}=3$.

Consider now the recorded current density-voltage-radiance (j−U−L) curves that are shown in Figure 3b. In the figure, the j−U and L−U data are marked by squares and solid lines, respectively. As seen, for the given structures, both kinds of characteristics are rather typical for OLEDs, with similar turn-on voltages, respectively, of circa 18 V and 16 V for the double-layer, PVK:Ir complex:OXD7/PFO-PEG, configurations with Ir(iqbt)_2_dpm and Ir(iqbt)_3_ emitters. The turn-on voltage for the PVK/PFO-TFP:Ir(iqbt)_3_: TPD/PFO-PEG (the three-layer) stack is significantly lower, at about 11 V, and is hence assigned to the more sophisticated device architecture with the thinnest emitting layer. Indeed, in the three-layer system, the films of the dedicated hole, as well as electron transporters, are used. This, together with the favorable mutual position of the corresponding HOMO and LUMO levels, provides the effective carrier injection into the NIR emitters. Such an OLED also gives the highest radiance value of max. 5 W/sr·m^2^ at current densities of about 250 mA/cm^2^. A similar value of L was found at j≈70 mA/cm^2^ in the case of the PVK:Ir(iqbt)_2_dpm: OXD7/PFO-PEG (the double-layer) device, but the poorest performance (L≈0.8 W/sr m^2^ at j≈20 mA/cm^2^) was measured for the two-layer OLED with the Ir(iqbt)_3_ emitter. Finally, from the j−U characteristics plotted in double-logarithmic scales, the values of the dimensionless parameter of trap distributions in emitting materials can be determined, which is necessary to investigate the performance of the Tq approach for the EQE experimental outcomes. Regarding the slopes, as in the inserts of Figure 3b (confer the Equation (7)), one obtains l=7.2 for the PVK:Ir(iqbt)_2_dpm:OXD7/PFO-PEG OLED structure, l=8.7 for the analogous system (but with Ir(iqbt)_3_ as the NIR emitter) and l=5.7 in the case of the PVK/PFO-TFP:Ir(iqbt)_3_:TPD/PFO-PEG configuration. The results of the EQE measurements are shown in Figure 4. As can be seen, the applied device architectures made it possible to obtain relatively high values of EQE. In particular, as shown in Figure 4a,b, EQEs as high as circa 3% and 1.5% were recorded in the case of the double-layer structures with Ir(iqbt)_2_dpm and Ir(iqbt)_3_ emitters, respectively, whereas an EQE of 0.9% was measured for the three-layer, PVK/PFO-TFP:Ir(iqbt)_3_: TPD/PFO-PEG, device. For all systems, the monotonic efficiency roll-off is clearly seen as following the initial, nearly flat section of the characteristics in the range of low current densities. Although the shape of the EQE-j dependencies remains rather unaffected by the OLED architecture and emitting compound, the efficiency roll-off seems to be structure-dependent, i.e., at 37% and 15% for the double-layer systems (Figure 4a,b), as well as at 29% in the case of the three-layer configuration from Figure 4c.

Let us analyze the performance of the theoretical approaches for rationalizing the EQEs. For this purpose, the EQE experimental outcomes have been fitted using the appropriate number of parameters. The list of relevant ones, together with the values of the corresponding rate constants obtained from reproduction procedures, are collected in Table 1 for each type of OLED. For the triplet-triplet annihilation (TTA) and the triplet-polaron quenching (Tq) models, the calculations were performed using the thicknesses of the recombination zone, w, as determined from the thickness of the emission layer. The exciton lifetimes, τ=2.0 μs for Ir(iqbt)_2_dpm and τ=2.9 μs for Ir(iqbt)_3_ emitters, were used as taken from the frozen 2-MeTHF glassy matrix [10]. The values of the current density, $j_{0}$, were individually adjusted to obtain the best fits for the experimental data. Regarding the electric-field-induced quenching (Onsager) approach, the initial intrapair radius, $r_{0}/r_{c}=0.1$, was used in calculations, as resulting from our previous Onsager studies [28]. In that paper, particularly, the $r_{0}/r_{c}\approx 0.1$ was utilized to reproduce the charge photogeneration measurements in some optoelectronic organic materials and was found to remain in good agreement with crystallographic data for a variety of compounds, including the iridium complex, fac-Ir(ppy)_3_. Note that $r_{0}/r_{c}=0.1$ implies that $r_{0}$ = 1.9 nm for T=298 K and $\varepsilon_{r}=3$.

The outcomes of the relevant procedures are shown in Figure 4, with points for the measured EQEs, the solid lines representing the TTA, the dashed ones the Tq and the dotted ones the Onsager model.

As seen from the figure, the TTA-curves do satisfactorily follow the EQEs that were recorded, with some upward-deviations increasing for higher current densities, particularly in the case of the three-layer OLED (confer Figure 4c). Therefore, the efficiency roll-off seems to be controlled mainly by the triplet-triplet exciton annihilation. The device-dependent values of the corresponding bimolecular rate constant, $\gamma_{\mathrm{TT}}$, as high as $12\times 10^{-12}$ cm^3^/s, $9\times 10^{-12}$ cm^3^/s and $0.5\times 10^{-12}$ cm^3^/s, were determined from Equations (2) and (3) during the reproduction procedure. Less satisfactory results were obtained for the Tq approach, where the nearly straight lines only roughly approximate the course of EQEs for all investigated OLEDs, with the second-order triplet-polaron interaction rate constant, $\gamma_{\mathrm{Tq}}$, of $\left(2.5-3\right)\times 10^{-12}$ cm^3^/s being calculated from Equation (8). Thus, the EQE roll-off effect seems rather to not be controlled solely by the triplet-polaron quenching mechanism for all types of devices. Similarly, the dominant operation of the electric-field-induced electron-hole pairs dissociation (confer Equations (9) and (10)) should rather not be taken into consideration, which remains in contradiction with previous studies on Ir(ppy)_3_-based green-light-emitting OLEDs, where the Onsager mechanism was suggested to operate [22]. Indeed, the relevant curves (marked by dotted lines in Figure 4) resemble the experimental outcomes only in the range of initial, low, current densities, with significant upward-deviations gradually increasing with j. These discrepancies may be ascribed to the Joule heat caused by the Ohmic losses and generated during charge injection and transport in operating OLED structures. A corresponding model was developed in the literature in which the heat-induced dissociation of excitons into free charge carriers is additionally introduced into the exciton-exciton annihilation mechanism (see paper [29] and the corresponding comment correcting the kinetics of the process [30]). Such a hypothesis seems to be justified, since relatively high voltages and current intensities are used in the regions of deviation from Figure 4. Moreover, the NIR-emitting compounds can be easily deactivated by heat. Some care is, however, advised to avoid a false interpretation of experimental data, since the influence of Joule heat was found to not be enough to affect the roll-off characteristics for small-area, low-brightness devices [29]. The more pronounced discrepancy seen for the three-layer OLED (confer Figure 4c) can in turn be ascribed to the emission of light by PVK forming the hole transporting layer. This emission results from the electric-field-induced lowering of the potential barrier for electrons at the interface between the PVK hole transporting and the emitting layer.

Following the obtained results, a clear conclusion about the origin of the roll-off effect is rather difficult to draw. First, we shall note that the limited performance of the approaches from Figure 4, especially those seen for high electric field ranges, could be assigned to the coexistence of various mechanisms that are not represented in the considered simple models, as well as some other factors regarding the OLED structures. In particular, the thicknesses of the recombination zones may be significantly overestimated from those of the device emissive layer in which the triplet states do interact. In fact, a diffusion length of excitons as high as 2–10 nm was determined in [Ir(ppy)_3_] guest-host systems [20] and [Ir(ppy)_3_]-cored dendrimers (Ir(ppy)_3_ = tris(2-phenylpyridine) iridium(III)) with phenylene- and carbazole-based dendrons, depending on the dendron size [31]. Next, the exciton annihilation (or quenching) processes may occur not only with the participation of triplet states in the phosphorescent molecules but also of those generated in the host matrix. Finally, the rather sophisticated architecture of the three-layer OLED with different conductivities of subsequent films may be responsible for the poor reproduction of the EQE from Figure 4c.

Regarding the issue of a possible coincidence of different mechanisms determining the efficiency roll-off, we shall recall the report in [20], where a unified model was presented, gathering both triplet-triplet annihilation and triplet-polaron quenching. For that approach, a single-rate equation was developed, assuming a nearly equal, strong roll-off-contribution of the TTA and Tq mechanisms. The resulting formula for EQE as a function of the current density involves both rate constants together with the phosphorescence lifetime and the thickness of the exciton formation zone. In this way, however, only the latter two factors are left as free-fitting parameters.

Apart from the annihilation/quenching processes, the population of OLED emitting states can depend on the current density just at the initial stage of exciton generation as a result of changes in the charge carrier balance. This process in stack-layer devices is due to differences between the structure energy barriers for the injection of electrons and holes, these barriers becoming easier to overcome for higher OLED currents/voltages or being in some cases electric-field-dependent [32], which in turn induces a relevant increase or decrease in the external quantum efficiency. A change in the charge balance induced by an increasing current density can also modify the localization or shape of the recombination zone, which provides another possibility of affecting the efficiency roll-off, hence by modulation of the outcoupling efficiency. The light outcoupling factor of an OLED represents a ratio between the number of photons emitted outside a given device and that of photons generated inside the structure. Its value is therefore determined by differences in the refractive indexes of stack compounds, surrounding air and other optical factors including the properties of the recombination zone. Thus, the outcoupling efficiency may become a function of the device voltage in some cases, and corresponding changes in the external quantum efficiencies may be observed. Changes in the charge balance are, however, difficult to incorporate reliably into a representative theoretical description, especially one regarding the complex multi-layer structures of OLEDs [32].

To sum up, in systems with phosphorescent emitters, the triplet-triplet annihilation mechanism together with the charge carrier imbalance are regarded in the literature as prevailingly influencing the efficiency roll-off. However, it should be noted here that the relative importance of other processes should not be definitely excluded due to their dependence on the properties of particular materials and the specific device structure used in a given OLED. Furthermore, an apparent roll-off effect can be detected as being induced by device degradation caused at sufficiently high current densities occurring even during one measuring cycle. Nevertheless, despite a gradual decrease in the external quantum efficiency for low current intensities due to a degradation process, the shape of corresponding experimental roll-off curves usually remains unaffected [33].

The decrease in the overall luminance over time during continuous driving is caused by the intrinsic degradation of an OLED. It is believed that this type of degradation is mainly due to the deterioration of organic (or metalorganic) molecules in the device [34]. The location and nature of these chemical degradation sites is highly dependent on the specific properties of the materials used (purity, morphology, reactivity), as well as the device structure, which is detrimental to the stability and reproducibility of the devices. Due to the presence of excitons, the problem is even more complicated in the emitting layer and at its interfaces with the adjacent transporting layers. In particular, bimolecular annihilation reactions involving excitons (like TTA or Tq processes in phosphorescent devices) lead to the population of “hot” excited states, which can enhance the formation of luminescence quenchers and non-radiative recombination centers. Although the low abundance of these entities in the organic layers is a real challenge for their detection, a number of techniques have been successfully employed, including ultraviolet and visible (UV-VIS), infrared absorption or Raman scattering, as well as nuclear (or electron) magnetic resonance spectroscopy [34,35]. A recently developed method, the imaging of real devices with super-resolved Raman spectroscopy, can also be implemented in this field [36]. It is also worth paying attention to the employing of laser desorption/ionization mass spectrometry (LDI-MS). For example, comparing the LDI-MS spectra of pristine and driven electrophosphorescent devices, it could be shown that cyclometalated homo- and heteroleptic iridium emitters can undergo ligand dissociation reactions during device operation [35,37].

Now, we shall discuss the values of the rate constants of triplet exciton annihilation. As determined via our fitting procedures, these constants are in the range of $\gamma_{\mathrm{TT}}=\left(0.5-12\right)\times 10^{-12}$ cm^3^/s, with the upper limit being rather too high for disordered organic systems. Indeed, bimolecular rate constants of a similar order, $\gamma_{\mathrm{TT}}=2\times 10^{-11}$ cm^3^/s and $\gamma_{\mathrm{TT}}=3.5\times 10^{-12}$ cm^3^/s, were measured, for instance, in anthracene and naphthalene crystals [21]. Here, we shall refer again to the issue of the recombination zone thickness, w, (confer Equations (2) and (3)) and note that reducing its width, e.g., by two times, would lower the $\gamma_{\mathrm{TT}}$ by the same factor. On the other hand, a variety of analogous results are known from the literature, but for other OLED phosphorescent emitters, where, generally, the $\gamma_{\mathrm{TT}}$ values do depend on the host matrix. This is the case for one of the most successful green triplet-emitters, Ir(ppy)_3_, for which a $\gamma_{\mathrm{TT}}$ in the range of $\left(1-3\right)\times 10^{-12}$ cm^3^/s was reported [38], with the lowest value for TPD-free Ir(ppy)_3_-doped polycarbonate and the highest one for high-TPD solid films or neat-emitter ones. A similar value of $\gamma_{\mathrm{TT}}=\left(3\pm 2\right)\times 10^{-12}$ cm^3^/s for samples of Ir(ppy)_3_ mixed by thermal coevaporation with 4,4’,4’-tris (N-carbazolyl)-triphenylamine (TCTA) was estimated both from time-resolved PL experiments and upon analysis of the EQE-j curves, assuming a recombination zone width w=10 nm and triplet lifetime τ=1.1 μs [20]. A somewhat wider, dendron-size-dependent range of $\gamma_{\mathrm{TT}}=\left(0.05-2\right)\times 10^{-12}$ cm^3^/s was determined for [Ir(ppy)_3_]-cored dendrimers [31]. Relatively high values for $\gamma_{\mathrm{TT}}$ of the order of $10^{-10}$ cm^3^/s were recorded for Ir(ppy)_3_ investigated as the neat film, in a 4,4’-N,N’-dicarbazole-biphenyl one and in a polystyrene matrix [39]. About two orders of magnitude of difference in the values of $\gamma_{\mathrm{TT}}$ observed for Ir(ppy)_3_ emitters can be rationalized in terms of a higher degree of molecular aggregation involved in samples prepared by spin-coating [39] in comparison to those that are vacuum-evaporated [20]. As for another emitter, the mutual annihilation of separately monomeric and dimeric exciton triplet states in neat platinum octaethyl-porphyrin (PtOEP) films was concluded in [40], and the corresponding rate constants, $\gamma_{\mathrm{TT}}≅8\times 10^{-12}$ cm^3^/s and even $\gamma_{\mathrm{TT}}≅8\times 10^{-15}$ cm^3^/s, were given. For the same emitter, but one embedded in an Alq_3_ matrix, a certain range of $10^{-13}-10^{-12}$ cm^3^/s has been reported [19]. Following such a variety of results, one may then conclude that annihilation events do occur between the triplet states in the host matrix, the emitter itself, as well as the host-guest ones. The interaction between the triplet states of a matrix should not, however, be a general rule. This is the case, for instance, for our OLED structure containing Ir(iqbt)_3_ molecules and a PVK-OXD7 host with a relatively high energy of triplet states in PVK and OXD7 equal to, respectively, 3 eV [41] and 2.7 eV [42], and, therefore, the interaction between the triplets of iridium emitters and the host matrix is less probable. It is worth noting here that despite some drawbacks of the OLEDs efficiency roll-off reproduction using a simple approach of triplet-triplet exciton annihilation, the order of the estimated relevant rate constant is rather acceptable. In fact, the value of $\gamma_{\mathrm{TT}}$ is roughly twice as large as that of $\gamma_{\mathrm{Tq}}$, the latter one representing the interaction of mobile triplet excitons with immobile charge carriers. On the other hand, since for the incoherent fully diffusion-controlled interaction $\gamma_{\mathrm{TT}}\approx 8{\pi D}_{T}R$ with $D_{T}$ being the diffusion coefficient of triplet excitons and R being the triplet-triplet interaction radius, assuming that R=10 Å [3,21] and for $\gamma_{\mathrm{TT}}=9\times 10^{-12}$ cm^3^/s (taken from Table 1), one obtains $D_{T}\approx 3.6\times 10^{-6}$ cm^2^/s. Such a value seems to be rational when compared with the diffusion coefficients of $1.5\times 10^{-4}$ cm^2^/s, $1.2\times 10^{-4}$ cm^2^/s and less than $0.2\times 10^{-4}$ cm^2^/s, respectively along the aa, bb and c′c′ crystallographic axes, which are known from the literature on anthracene crystals [21].

## 4. Conclusions

In this paper, we have measured and analyzed the efficiency roll-off in NIR-emissive OLEDs based on Ir(iqbt)_2_(dpm) and Ir(iqbt)_3_ complexes. The double-layer solution-processed structure with emitters embedded in the PVK-OXD7 host matrix, as well as the analogous three-layer one extended by a carrier-conducting PVK film, were investigated. The device-dependent values of the external QE (up to circa 3%) with a moderate quantum efficiency roll-off were recorded. The current density-external QE characteristics of the structures were analyzed in comparison to the predictions from three theories on the roll-off effect. We considered the triplet-triplet annihilation, the triplet-polaron quenching, as well as the Onsager model of the electron-hole pair dissociation as responsible for the emitting state quenching. Following the fitting procedure, the roll-off effect seems to be controlled mainly by the triplet-triplet exciton annihilation, with the interaction between triplets of iridium emitters and the host matrix found as being less probable. The relevant rate constant was found to be $\left(0.5-12\right)\times 10^{-12}$ cm^3^/s, and, as compared with the rate constants $\gamma_{\mathrm{TT}}=2\times 10^{-11}$ cm^3^/s and $\gamma_{\mathrm{TT}}=3.5\times 10^{-12}$ cm^3^/s for anthracene and naphthalene crystals, we consider the determined values as rather too high for disordered organic systems, which is assigned to the simplicity of the applied model. We therefore infer the coexistence of some other mechanisms, ones, however, with a less significant contribution to the overall emission quenching.

## Figures and Tables

**Table 1:** The values of the parameters used for fitting the OLED EQEs’ data as well as the values of the Tq and TTA rate constants from the reproduction procedures.

EL Diode	d (nm)	w (nm)	τ (μs)	l	γ_Tq_ (10^−12^ cm^3^/s)	j_0_ (A/cm^2^)	γ_TT_ (10^−12^ cm^3^/s)
PVK:Ir(iqbt)_2_dpm: OXD7/PFO-PEG	269	204	2	7.2	3	0.28	12
PVK:Ir(iqbt)_3_: OXD7/PFO-PEG	266	201	2.9	8.7	2.5	0.18	9
PVK/PFO-TFP:Ir(iqbt)_3_: TPD/PFO-PEG	189	79	2.9	5.7	2.5	1.37	0.5
